# Metabolic Profiling Provides a System Understanding of Hypothyroidism in Rats and Its Application

**DOI:** 10.1371/journal.pone.0055599

**Published:** 2013-02-07

**Authors:** Si Wu, Guangguo Tan, Xin Dong, Zhenyu Zhu, Wuhong Li, Ziyang Lou, Yifeng Chai

**Affiliations:** 1 School of Pharmacy, Second Military Medical University, Shanghai, China; 2 Shanghai Key Laboratory for Pharmaceutical Metabolite Research, Shanghai, China; 3 The 22^nd^ Hospital of Chinese People's Liberation Army, Geermu, China; Universidade Federal do Rio de Janeiro, Brazil

## Abstract

**Background:**

Hypothyroidism is a chronic condition of endocrine disorder and its precise molecular mechanism remains obscure. In spite of certain efficacy of thyroid hormone replacement therapy in treating hypothyroidism, it often results in other side effects because of its over-replacement, so it is still urgent to discover new modes of treatment for hypothyroidism. *Sini* decoction (*SND*) is a well-known formula of Traditional Chinese Medicine (TCM) and is considered as efficient agents against hypothyroidism. However, its holistic effect assessment and mechanistic understanding are still lacking due to its complex components.

**Methodology/Principal Findings:**

A urinary metabonomic method based on ultra performance liquid chromatography coupled to mass spectrometry was employed to explore global metabolic characters of hypothyroidism. Three typical hypothyroidism models (methimazole-, propylthiouracil- and thyroidectomy-induced hypothyroidism) were applied to elucidate the molecular mechanism of hypothyroidism. 17, 21, 19 potential biomarkers were identified with these three hypothyroidism models respectively, primarily involved in energy metabolism, amino acid metabolism, sphingolipid metabolism and purine metabolism. In order to avert the interference of drug interaction between the antithyroid drugs and *SND*, the thyroidectomy-induced hypothyroidism model was further used to systematically assess the therapeutic efficacy of *SND* on hypothyroidism. A time-dependent recovery tendency was observed in *SND*-treated group from the beginning of model to the end of treatment, suggesting that *SND* exerted a recovery effect on hypothyroidism in a time-dependent manner through partially regulating the perturbed metabolic pathways.

**Conclusions/Significance:**

Our results showed that the metabonomic approach is instrumental to understand the pathophysiology of hypothyroidism and offers a valuable tool for systematically studying the therapeutic effects of *SND* on hypothyroidism.

## Introduction

Hypothyroidism is a condition in which the body lacks sufficient thyroid hormone. The thyroid hormone plays a fundamental role in the development, differentiation, proliferation and physiology of all cells in an organism [1], [2]. Therefore, inadequate thyroid hormone has widespread consequences for the body. Some studies have shown that hypothyroidism may induce delayed skeletal development [3], cardiovascular diseases [4], secondary hypertension [5], deterioration of human reproductive health [6], and changes in the brain structure and function [7].

The main treatment for hypothyroidism at present is thyroid hormone replacement therapy [8], [9]. However, due to difficulties in controlling the level of thyroid hormones through use of drugs or an exogenous source of thyroid hormone, although patients may experience partial relief of the symptoms, they usually suffer over-replacement which may result in an increased risk of cardiovascular disease [10], osteoporosis [11] and subclinical liver damage [12]. Therefore, there is an urgent need to seek new strategies for the management of hypothyroidism.

Traditional Chinese Medicine (TCM), a unique medical system with the significant characteristics of pursuing an overall therapeutic effect with a multi-target treatment, attempts to improve therapeutic efficacy and reduce drug-related adverse effects at the same time [13], [14]. According to TCM theory, the essential pathogenesis of hypothyroidism is “Kidney-*Yang* Deficiency Syndrome” [15], [16]. Here, kidney is not only the urinary organ, but it is regarded as the root of life activities and represents the origin of our congenital or inherited foundation [17]. To some extent, “Kidney-*Yang* Deficiency Syndrome” represents a condition of holistic exhaustion of the whole body. Modern medicine research indicates that “Kidney-*Yang* Deficiency Syndrome” is a functional disorder with varying degrees of hypothalamic-pituitary-target gland (thyroid, adrenal and gonad) axis [18], [19]. *Sini* decoction (*SND*), a well-known formula for invigorating *Yang*, is officially recorded in Chinese pharmacopoeia (2010 edition). It consists of *Acontium carmichaeli*, *Glycyrrhiza uralensis* and *Zingiber officinate*, and is mainly applied for “Kidney-*Yang* Deficiency Syndrome” [20], [21] and cardiovascular disease [22], [23]. Our previous studies have successfully validated the therapeutic effect of *SND* on cardiovascular disease [24], [25]. However, it is still lacking of the evaluation of the holistic and synergistic efficacy of *SND* on hypothyroidism due to the complexity of hypothyroidism and the active compounds in *SND*.

This dilemma calls upon the debut of metabonomics at the end of last century. The metabonomic approach as a top-down and nonhypothesis-driven analysis holds the promise of a non-discriminatory analysis of metabolic biomarkers that could elucidate the pathogenesis of diseases and help monitor therapeutic response [26]. The systemic thinking of metabonomics and its aim at grasping integral function make it pillars of potential bridge between TCM and western medicine, which may beneficially provide an opportunity to open up the black box between evidence-based Chinese medicine and the organism [27]. To date, there has been wide applications of metabonomic approach in the field of TCM [28], [29], which strive to illustrate the efficacy and mechanism of ancient TCM on modern diseases and make further efforts to develop new drugs for complex diseases from the insight of TCM theory.

Van Ravenzwaay's group [30]–[32] successfully employed standardized test protocols and metabolomics to discover new biomarkers and specific patterns in response to propylthiouracil (PTU)- and methimazole (MMI)- induced perturbations in rat plasma. Maria Klapa's group [33] applied metabolomic method to delineate significant alterations in cerebellar metabolic physiology related to hypothyroidism. However, little is known about the overall changes of urinary metabolites in an organism with hypothyroidism. In the present study, a UHPLC/TOF-MS based urine metabolomic approach was employed to characterize the status of thyroid dysfunction in three widely used rat models of MMI-, PTU- and thyroidectomy-induced hypothyroidism. In addition, in order to avoid the potential interference of drug interaction between antithyroid substances (MMI and PTU) and *SND*, we used the thyroidectomy-induced hypothyroid model to further investigate the protective effects of *SND* on hypothyroidism.

## Results

### Effects of hypothyroidism on the Body Weight (BW) time profiles

Before the hypothyroidism models were established, there was no significant difference in body weight between control group (172.1±3.2 g), MMI group (171.4±2.9 g) and PTU group (173.5±3.4 g). However, body weight was markedly lower in MMI group (194.7±2.8 g; *p*<0.001) and PTU group (203.2±3.1 g; *p*<0.001) than in control group (251.2±3.6 g) after the antithyroid drug-induced hypothyroidism models were established.

**Figure 1:**
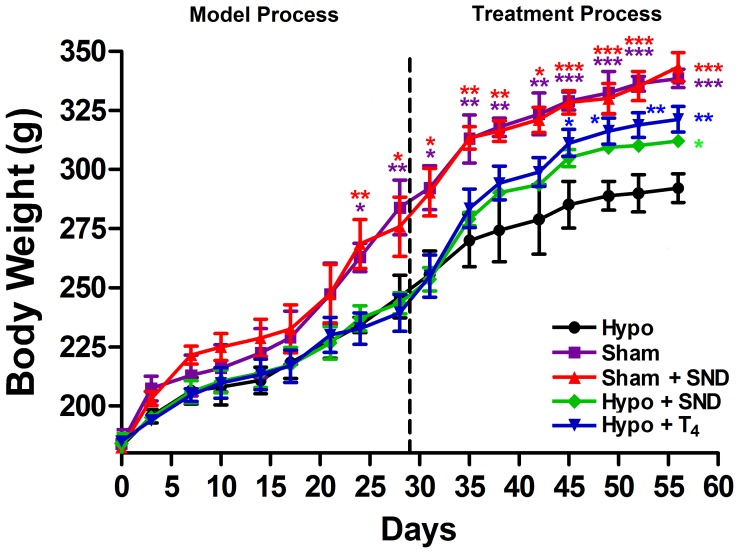
The Body Weight (BW) profiles during the model and treatment periods. (box represents Sham group, triangle represents Sham + *SND* group, dot represents Hypo group, diamond represents Hypo + *SND* group, inverted triangle represents Hypo + T_4_ group).

The BW time profiles for surgical groups during the animal study period are shown in **Figure 1**. There were no differences among these five groups before surgery (Day 0). During the thyroidectomy-induced hypothyroidism period (Day 1–Day 28), BW in the Sham and Sham + *SND* groups kept increasing sharply, while BW in the Hypo, Hypo + *SND* and Hypo + T_4_ groups followed a slowly increasing compared with the Sham and Sham + *SND* groups. During the treatment period (Day 29–Day 57), BW remained rising quickly in the Sham and Sham + *SND* groups and no significant difference between Sham and Sham + *SND* groups was observed, while BW in the Hypo group received a more slowly increase, and the BW in the Hypo + *SND* and Hypo + T_4_ groups showed an ascending trend compared with the Hypo group. In addition, the increasing trend is more distinct in Hypo + T_4_ group than in Hypo + *SND* group.

### Biochemical analysis

To validate the establishment of the animal model and the effects of *SND* and T_4_ on hypothyroidism, serum T_3_ and T_4_ levels were determined. The results in surgical groups are given in **Table 1**. The T_3_ and T_4_ concentrations decreased significantly 4 weeks after surgery in the Hypo, Hypo + *SND* and Hypo + T_4_ groups as compared to the Sham group. The numeral values of T_3_ and T_4_ in Hypo + *SND* and Hypo + T_4_ groups were obviously improved compared to the hypothyroid group after 4 weeks treatment. Meanwhile, there was no significant difference between Sham and Sham + *SND* groups in T_3_ and T_4_ concentrations throughout the experiment.

**Table 1:** Sample determination of total triiodothyronine (T_3_) and total thyroxine (T_4_) in rat serum for the surgery-induced hypothyroid groups (mean±S.D.)^a^ (n = 8).

Group	T_3_		T_4_	
	4w after Surgery	4w after Treatment	4w after Surgery	4w after Treatment
Sham group	0.48±0.06	0.46±0.05	47.66±5.13	44.35±4.22
Sham + *SND* group	0.46±0.09	0.47±0.06	45.52±3.75	46.71±4.52
Hypo group	0.29±0.04**	0.24±0.07**	20.06±2.30***	19.87±2.14***
Hypo + *SND* group	0.27±0.04**	0.33±0.04#	17.80±1.19***	26.40±1.10##
Hypo + T_4_ group	0.29±0.03**	0.43±0.03###	18.49±1.37***	35.18±1.22###

a The unit of T_3_ and T_4_ is ng/mL.

** *p*<0.01 (compared to Sham group),

*** *p*<0.001(compared to Sham group).

# *p*<0.05 (compared to Hypo group),

## *p*<0.01 (compared to Hypo group),

### *p*<0.001 (compared to Hypo group).

There were no differences in initial T_3_ and T_4_ concentrations among control, MMI and PTU groups, but after 4 weeks of antithyroid drugs treatment, the T_3_ and T_4_ levels of MMI group and PTU group were strikingly lower than that of the control group (shown in **Table S1**).

### Assessment of the health status

During the experimental period, rectal temperature, food intake and water intake were recorded before and after the hypothyroidism model established and after 4 weeks treatment in order to represent the health status of rats. The data in surgery- and antithyroid drug-induced hypothyroid groups were shown in **Table 2** and **Table S2**, respectively. The results indicated that both thyroidectomy and antithyroid drugs (MMI and PTU) led to significant decline of rectal temperature, food intake and water intake in hypothyroid groups. After 4 weeks treatment with *SND* and T_4_, the health status of the rats in the Hypo + *SND* and Hypo + T_4_ groups improved with different degrees, while the Hypo group without *SND* or T_4_ treatment was still in a restrained state and got worse. Comparing the data from Hypo + *SND* and Hypo + T_4_ groups after 4 weeks treatment; it is found that T_4_ seemed to be more efficient in restoring water intake as compared to *SND*, while *SND* showed better efficiency in increasing rectal temperature than T_4_. And food intake was recovered dramatically in Hypo + T_4_ group, while no difference was observed between Hypo and Hypo + *SND* groups.

**Table 2:** The physical variance for the surgery-induced hypothyroid groups (mean±S.D.) (n = 8).

Group	Rectal Temperature (°C)			Food Intake (g/day)			Water Intake (g/day)		
	Before Surgery	4w after Surgery	4w after Treatment	Before Surgery	4w after Surgery	4w after Treatment	Before Surgery	4w after Surgery	4w after Treatment
Sham group	37.8±0.2	37.9±0.3	37.8±0.1	24.56±2.12	25.56±1.97	25.96±2.08	24.87±2.02	24.83±2.11	25.05±1.87
Sham + *SND* group	37.7±0.1	37.8±0.2	37.9±0.2	24.43±1.96	25.81±2.03	26.12±2.17	24.68±1.89	24.91±2.09	25.13±2.10
Hypo group	37.9±0.3	37.4±0.3*	37.2±0.2**	24.82±2.17	19.02±1.94***	18.12±1.87***	24.89±1.98	22.79±2.07**	21.79±1.70***
Hypo + *SND* group	37.8±0.2	37.4±0.2*	37.7±0.1##	24.76±1.93	18.84±1.81***	19.64±2.01	24.78±2.10	22.85±1.98**	23.90±2.07##
Hypo + T_4_ group	37.7±0.2	37.5±0.3*	37.6±0.2#	24.49±2.01	19.14±2.07***	24.12±2.09###	24.74±1.92	22.81±2.10**	24.99±1.96###

* *p*<0.05,

** *p*<0.01,

*** *p*<0.001 compared to Sham group.

# *p*<0.05,

## *p*<0.01,

### *p*<0.001 compared to Hypo group.

### Urinary metabolic profiling by UHPLC-MS

**Figure 2:**
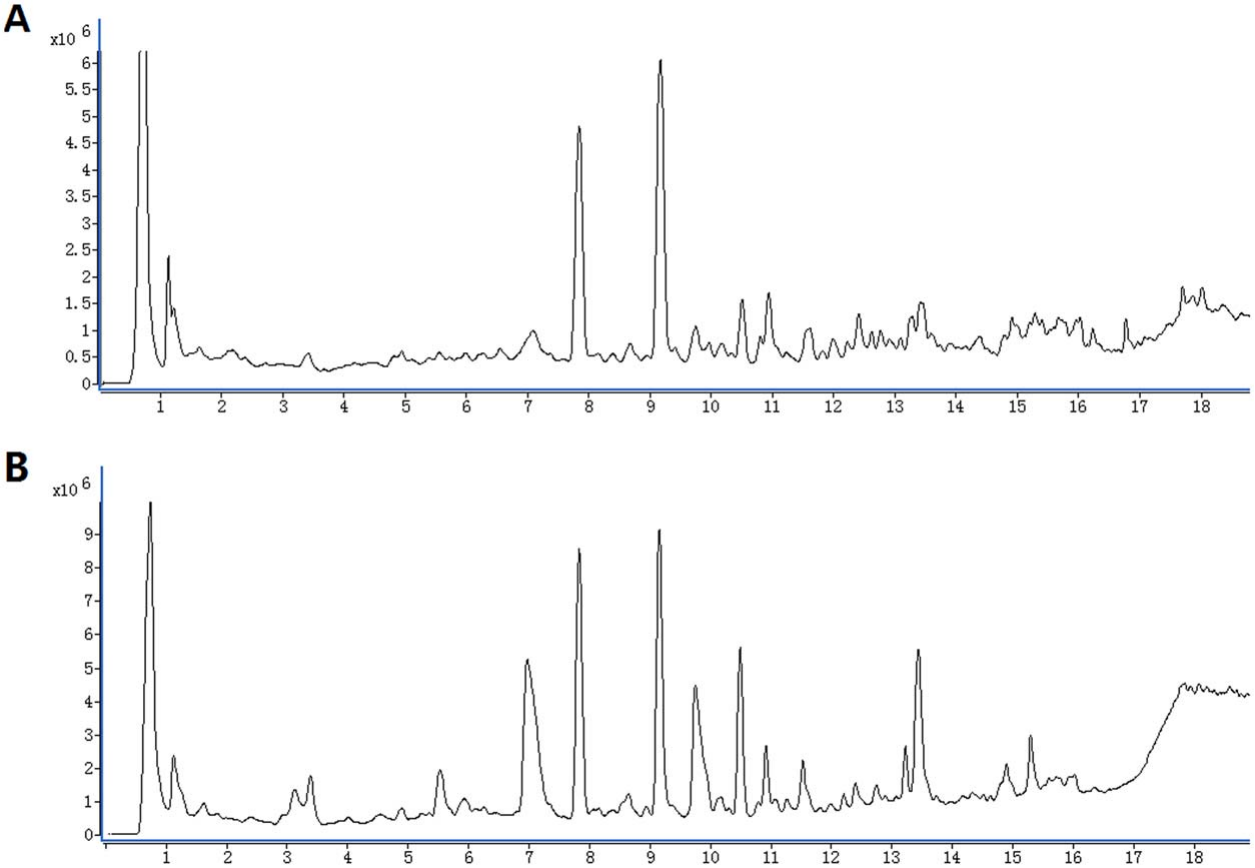
Typical urine total ion current (TIC) chromatograms in positive ionization mode (A), negative ionization mode (B).

Using the optimal UHPLC-Q-TOFMS condition described above, the representative total ion current (TIC) profiles of the urine samples in positive and negative ionization modes are shown in **Figure 2**. To validate the stability of the established method, an in-house QC sample was prepared by mixing an equal volume (100 µL) from each sample and injected every 8 samples during the whole sample analysis [34]. A subset of peaks across the QC samples taking account of intensities and retention time was examined. It was found that the relative standard deviation (RSD) was below 0.5% for the retention time, and below 6% for the peak area in both ionization modes. To evaluate the repeatability, seven parallel samples obtained from a random urine sample were extracted by the same extraction method and injected continuously. The same subset of peaks was tested and the results showed that the RSD was below 0.5% for the retention time, and below 5% for the peak area in both ionization modes. The detailed data of stability and repeatability are listed in **Table 3**. The results indicated that the method was reliable for the subsequent analysis.

**Table 3:** The result of stability and repeatability of the proposed method.

Mode	*m/z*	Stability (*n* = 7)				Repeatability (*n* = 7)			
		RT (min)		Peak area		RT (min)		Peak Area	
		Mean	RSD (%)	Mean	RSD (%)	Mean	RSD (%)	Mean	RSD (%)
ESI^+^	176.0682	13.42	0.05	209231	3.10	13.39	0.05	210740	2.33
	194.0776	9.16	0.27	13257140	1.20	9.15	0.14	13537762	2.32
	218.1030	11.88	0.08	148077	1.62	11.92	0.35	147275	1.39
	233.0876	10.93	0.06	1574021	3.53	10.90	0.07	1585795	1.37
	314.2310	15.53	0.40	129634	2.07	15.53	0.41	122482	1.41
	318.3008	15.96	0.28	705698	1.37	15.96	0.02	695028	3.53
	487.1570	10.94	0.07	110530	5.08	10.98	0.05	113077	3.32
ESI^−^	158.0820	6.26	0.12	2126790	3.26	6.28	0.18	1916201	3.35
	191.0190	1.21	0.48	3385916	3.89	1.23	0.45	3564837	4.23
	201.0199	13.42	0.06	13422607	1.79	13.43	0.09	13567883	2.19
	218.1028	4.92	0.14	965880	2.50	4.95	0.14	987233	1.92
	357.1077	15.30	0.03	2522596	1.18	15.29	0.03	2661158	0.89
	385.1460	9.15	0.07	5404326	1.20	9.18	0.11	5594287	0.98
	407.2760	16.43	0.04	5057190	1.39	16.40	0.44	524508	1.81

### Multivariate statistical analysis of metabolites

**Figure 3:**
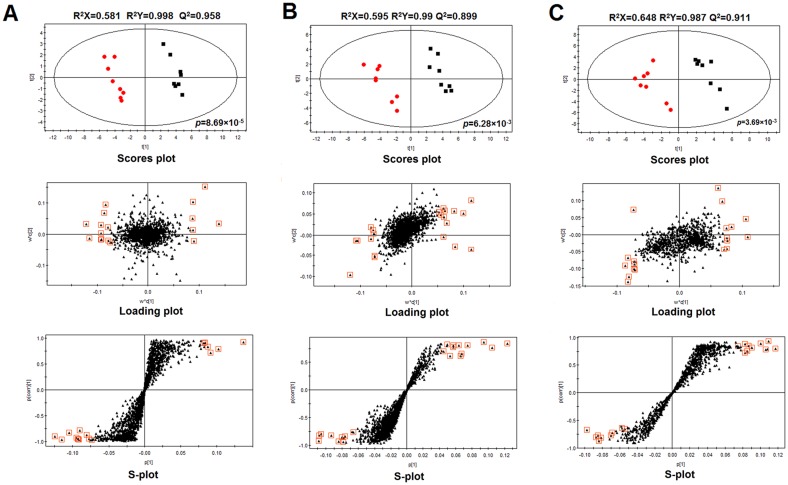
PLS-DA scores plot (top panel), loading plot (middle panel) and S-plot (bottom panel) of the UHPLC/TOF-MS spectral from control group (box) and hypothyroid group (dot). (**A**: control group *vs* MMI group; **B**: control group *vs* PTU group; **C**: control group *vs* thyroidectomy-induced hypothyroid group).

Before multivariate statistical analysis, peaks with a retention time less than 0.5 min (near to the dead time) were excluded due to a high degree of ion suppression that they suffered [35]. The datasets from positive and negative ionization modes contained 1018 and 1060 variables with control and MMI groups, the values were 899 and 1162 respectively with control and PTU groups, and the values were 957 and 909 with Sham and Hypo groups. The two datasets from positive and negative ionization modes were combined as a new dataset. To determine whether the metabolite fingerprints in urine differed between the control and hypothyroid groups in our metabonomic approach, we constructed partial least squares linear discriminant analysis (PLS-DA) models which had been widely used in metabonomic study [36], [37]. In this study, three PLS-DA models were established and employed to identify biomarkers which were related to MMI-, PTU- and thyroidectomy-induced hypothyroidism, which are showed in **Figure 3**. Commonly, R^2^Y provides an estimate of how well the model fits the Y data, whereas Q^2^Y is an estimate of how well the model predicts the Y [28]. In order to gain high predictive ability, the values of R^2^Y and Q^2^Y should be close to 1. The related parameters of the three PLS-DA models are given in **Figure 3**. As **Figure 3** shows, there is a distinguished classification between the clustering of the control and MMI groups (**Figure 3A**), the control and PTU groups (**Figure 3B**), as well as the Sham and Hypo groups (**Figure 3C**). The R^2^Y and Q^2^Y were all above 0.99 and 0.89 in the three models, respectively, which indicated that the models had good prediction characteristics.

### Detection and identification of biomarker candidates

To select potential biomarkers related to hypothyroidism, statistically significant differences for the variables between the Sham, Sham + *SND*, Hypo, Hypo + *SND*, Hypo + T_4_ groups were tested by ANOVA and the Tukey Post hoc test for comparisons of multiple groups. Metabolites that differed significantly between Sham and Hypo groups after correction for multiple comparisons (false discovery rate q<0.05) were identified as candidate biomarkers. Next, the corresponding loading plot and S-plot (shown in **Figure 3**) were used to further screen these candidate biomarkers, in which the ions furthest away from the origin contributed remarkably to the separation of the Sham and Hypo groups and may be regarded as potential biomarkers. Furthermore, the variable importance for projection (VIP) reflecting the importance of variables has been applied to filter the important metabolites in the model. In this study, metabolite ions with VIP value >1.5 were considered as potential biomarkers. Following the criterions above, 19 metabolite ions were selected as potential biomarkers related with thyroidectomy-induced hypothyroidism. With similar selection process, 17 and 21 metabolite ions were screened out as the biomarkers related with MMI- and PTU-induced hypothyroidism, respectively.

**Figure 4:**
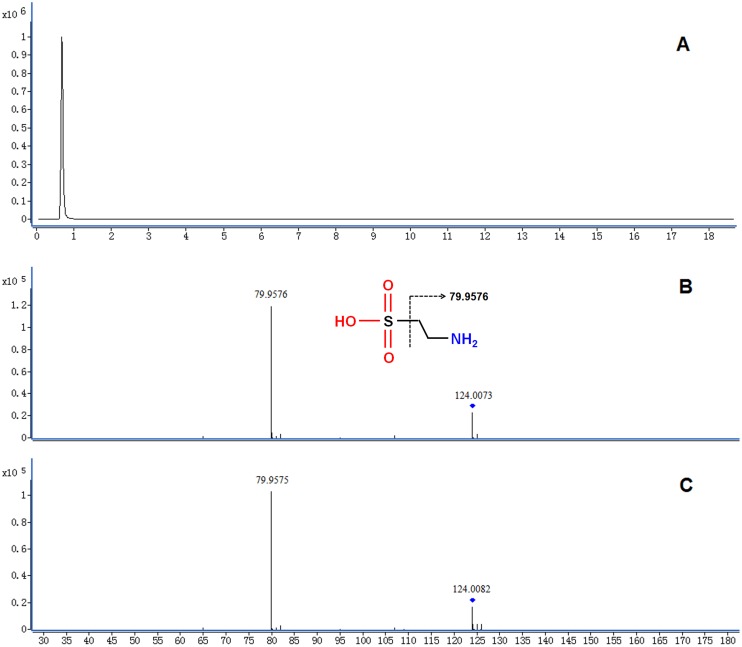
Identification of a selected marker (*m/z* 124.0082). (**A**) Extracted ion chromatogram (EIC) of m/z 124.0082 (t*_R_* = 0.69 min); (**B**) MS/MS spectrum of the ion in the urine; (**C**) MS/MS spectrum of a commercial standard. The collision energy was 18 V.

The detailed method for the compound identification was described in our previous work [25]. In brief, the corresponding quasi-molecular ion peak was found according to the accurate mass and retention time in the extracted ion chromatogram (EIC) and speculated the most probable molecular formula. **Figure 4A** shows the EIC of a typical ion whose *m/z* is 124.0073. Then, MS/MS analysis of *m/z* 124.0073 in urine was performed using UHPLC-Q-TOFMS in the same chromatographic and mass spectrometric conditions (**Figure 4B**). With its fragmentation information and the freely accessible databases, the fragment ion m/z 79.9576 was speculated to produce by the loss of –C_2_H_6_N. Therefore, the m/z 124.0073 was identified as taurine according to the elemental composition, retention time and fragmentation information. Finally, the MS/MS spectrum of the commercial standard taurine (**Figure 4C**) was used to confirm the identified compound. Similarly, 17, 21, 19 potential biomarkers related with MMI-, PTU- and thyroidectomy-induced hypothyroidism were identified and are listed in **Table 4**.

**Table 4:** Potential Biomarkers and their Metabolic Pathway.

Mode	NO.	RT(min)	Ion (*m/z*)	Identification	Trend^c^	Related Pathway	MMI vs Control			PTU vs Control			Hypo vs Sham		
							VIP^d^	FC^e^	*p* f	VIP	FC	*p*	VIP	FC	*p*
ESI^+^	1	0.59	146.1641	Spermidine^a^	**↓**	Arginine and proline metabolism	1.66	0.73	1.04E-03	—			—		
	2	10.93	233.1137	N_2_-Succinyl-L-ornithine^b^	**↑**	Arginine and proline metabolism	1.51	1.53	2.02E-02	2.36	1.92	9.07E-05	2.08	1.8	9.40E-05
	3	7.25	188.0342	Kynurenic Acid^a^	**↑**	Tryptophan metabolism	1.97	1.72	1.13E-03	2.86	2.22	8.72E-04	3.01	2.41	7.12E-05
	4	6.64	206.0426	Xanthurenic Acid^b^	**↑**	Tryptophan metabolism	2.35	1.73	6.12E-03	2.19	1.53	3.22E-03	2.52	1.82	8.92E-03
	5	7.59	134.0572	Indoxyl^b^	**↑**	Tryptophan metabolism	1.76	1.2	5.13E-03	1.98	1.87	1.29E-03	—		
	6	0.71	118.0663	Indole^b^	**↑**	Tryptophan metabolism	2.27	1.79	8.70E-04	2.15	1.71	1.01E-03	—		
	7	10.49	302.3059	Dihydrosphingosine^a^	**↓**	Sphingolipid metabolism	—			2.05	0.54	1.22E-03	—		
	8	15.97	318.3008	Phytosphingosine^a^	**↓**	Sphingolipid metabolism	1.85	0.69	8.31E-03	2.20	0.59	7.89E-04	2.61	0.41	9.87E-05
	9	0.68	137.0715	N-methylnicotinamide^b^	**↓**	Nicotinate and nicotinamide metabolism	—			1.71	0.84	4.32E-02	1.73	0.83	4.10E-02
	10	4.4	132.0775	Creatine^a^	**↓**	Energy metabolism	—			1.54	0.83	8.53E-03	—		
	11	0.71	114.0667	Creatinine^a^	**↑**	Energy metabolism	—			—			2.13	1.69	1.85E-02
	12	9.16	194.0802	Phenylacetylglycine^b^	**↑**	Phenylalanine metabolism	2.67	2.15	1.91E-05	1.90	1.58	4.86E-03	2.42	1.96	3.12E-05
	13	13.42	176.0682	Dopamine^b^	**↑**	Phenylalanine metabolism	2.15	2.13	9.80E-05	—			1.93	1.97	7.70E-05
	14	0.75	144.0778	Phenylethylamine^b^	**↑**	Phenylalanine metabolism	2.71	2.14	8.24E-04	—			—		
	15	0.65	188.1751	N_8_-Acetylspermidine^b^	**↑**	Related with Thyroid cancer	1.86	1.64	7.79E-03	—			—		
ESI^−^	16	1.58	117.0192	Succinic acid^a^	**↓**	Citrate cycle	1.97	0.66	4.72E-03	2.58	0.49	2.02E-04	—		
	17	0.84	145.0136	α-ketoglutaric Acid^a^	**↓**	Citrate cycle	—			2.22	0.61	7.96E-04	—		
	18	1.44	173.0089	cis-Aconitate^b^	**↓**	Citrate cycle	1.6	0.81	8.89E-03	—			1.72	0.72	8.30E-04
	19	1.02	191.019	Citric Acid^a^	**↓**	Citrate cycle	2.35	0.62	5.13E-03	1.70	0.71	2.72E-02	3.32	0.43	3.66E-04
	20	0.69	124.0073	Taurine^a^	**↓**	Taurine and hypotaurine metabolism	2.47	0.61	5.43E-04	1.80	0.73	4.76E-03	2.87	0.51	8.70E-05
	21	14.78	253.0494	5-L-Glutamyl-Taurine^b^	**↓**	Taurine and hypotaurine metabolism	—			1.78	0.76	7.60E-03	2.89	0.32	6.95E-05
	22	0.73	138.9702	Sulfoacetic Acid^b^	**↓**	Taurine and hypotaurine metabolism	—			1.52	0.79	7.91E-03	—		
	23	0.74	157.0362	Allantoin^a^	**↓**	Purine metabolism	—			1.83	0.69	3.12E-03	2.14	0.5	2.40E-05
	24	1.17	167.0205	Uric Acid^a^	**↑**	Purine metabolism	1.7	1.36	7.04E-04	1.51	1.25	1.50E-03	2.18	1.78	3.14E-05
	25	0.72	135.0295	Hypoxanthine^b^	**↓**	Purine metabolism	—			—			2.79	0.69	2.84E-05
	26	9.83	187.0031	Xanthine^b^	**↓**	Purine metabolism	—			—			2.26	0.64	3.56E-02
	27	7.85	178.047	Hippuric Acid^a^	**↑**	Phenylalanine metabolism	2.69	3.12	1.32E-03	2.83	3.78	9.57E-04	2.31	2.72	3.18E-03
	28	7.01	181.0498	Homovanillic Acid^a^	**↑**	Phenylalanine metabolism	—			1.60	1.42	7.68E-03	1.56	1.39	9.32E-03
	29	9.88	187.0719	N-Acetyl-L-Glutamide^b^	**↓**	A source of Glutamine	—			2.05	0.61	5.79E-03	1.66	0.73	3.89E-02

a Metabolites identified by retention time and MS^2^ spectrum of an authentic standard;

b Metabolites putatively annotated.

c “↑” and “↓” represent the compound is up- and down-regulated in model groups (MMI, PTU and Thyroidectomy-induced hypothyroid groups) compared with the control and sham groups, respectively.

d VIP, variable importance on projection. VIP values ≥1.5 are considered significantly changed metabolites.

e The fold change (FC) of relative amounts of the model groups (MMI, PTU and Thyroidectomy-induced hypothyroid groups) compared with the control and sham groups, respectively.

f The *p* value was calculated by one-way ANOVA and then by the Tukey post hoc test for comparisons of multiple groups (after correction for multiple hypothesis testing and false discovery rate<0.05).

### Effects of *SND* treatment

**Figure 5:**
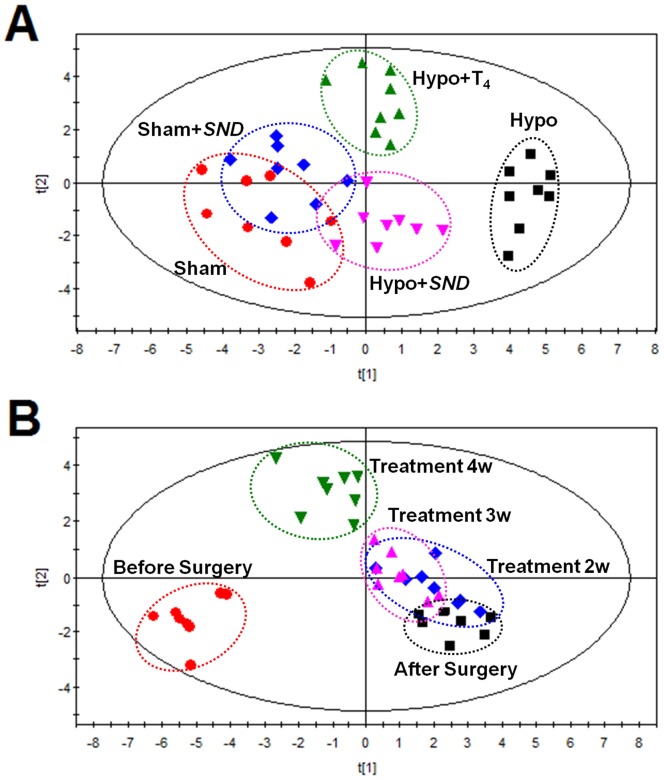
PLS-DA scores plot to evaluate the therapeutical effects of SND. (**A**) PLS-DA scores plot derived from urine levels of 21 potential biomarkers in Sham group (dot), Sham + *SND* group (diamond), Hypo group (box), Hypo + *SND* group (inverted triangle) and Hypo + T_4_ group (triangle). (**B**) A time-related trajectory of metabolite patterns from PLS-DA model classifying the state of rats at different time points: before surgery (dot), after surgery (box), treatment for two weeks (diamond), treatment for three weeks (triangle); treatment for four weeks (inverted triangle).

The thyroidectomy-induced hypothyroidism model was further used to evaluate the therapeutic effects of *SND* and T_4_ on hypothyroidism. A PLS-DA model based on the selected 19 related potential biomarkers was built. The R^2^Y and Q^2^ (cum) of the newly established PLS-DA model were 0.972 and 0.763 respectively, indicating that the model had a great ability of fitness and prediction. As shown in **Figure 5A**, a separation of the Sham and Hypo groups was clearly achieved, while the Hypo + *SND* and Hypo + T_4_ group was mainly located between the Sham and Hypo group, indicating that both *SND* and positive drug T_4_ had different degrees of protective effects on hypothyroidism. Meanwhile, Sham + *SND* group shared much overlap with Sham group, which demonstrated that there is no obvious difference between Sham and Sham + *SND* groups in metabolic patterns.

To better understand the time-related trajectory of the metabolic pattern with *SND* treatment from the very beginning of established hypothyroid model to the end of treatment, a score plot of PLS-DA map from the Hypo + *SND* group at various time points was illustrated in **Figure 5B**. In the map, each spot represented a sample, and each assembly of samples indicated a particular metabolic pattern at different time points. The starting spots of the Hypo + *SND* group before surgery obviously deviated from those after surgery, suggesting that high fluctuations occurred in the metabolic regulatory network after thyroidectomy. Furthermore, the spots of the Hypo + *SND* group at week 2 and 3 of treatment clustered near the starting spots gradually, showing a backward tendency towards the normal status before surgery, which might be an indication of the accumulated effect of *SND*. The last spots of the Hypo + *SND* group at week 4 of treatment ultimately approached the initial state, suggesting that *SND* showed recovering effects on the rats with hypothyroidism to some extent.

**Figure 6:**
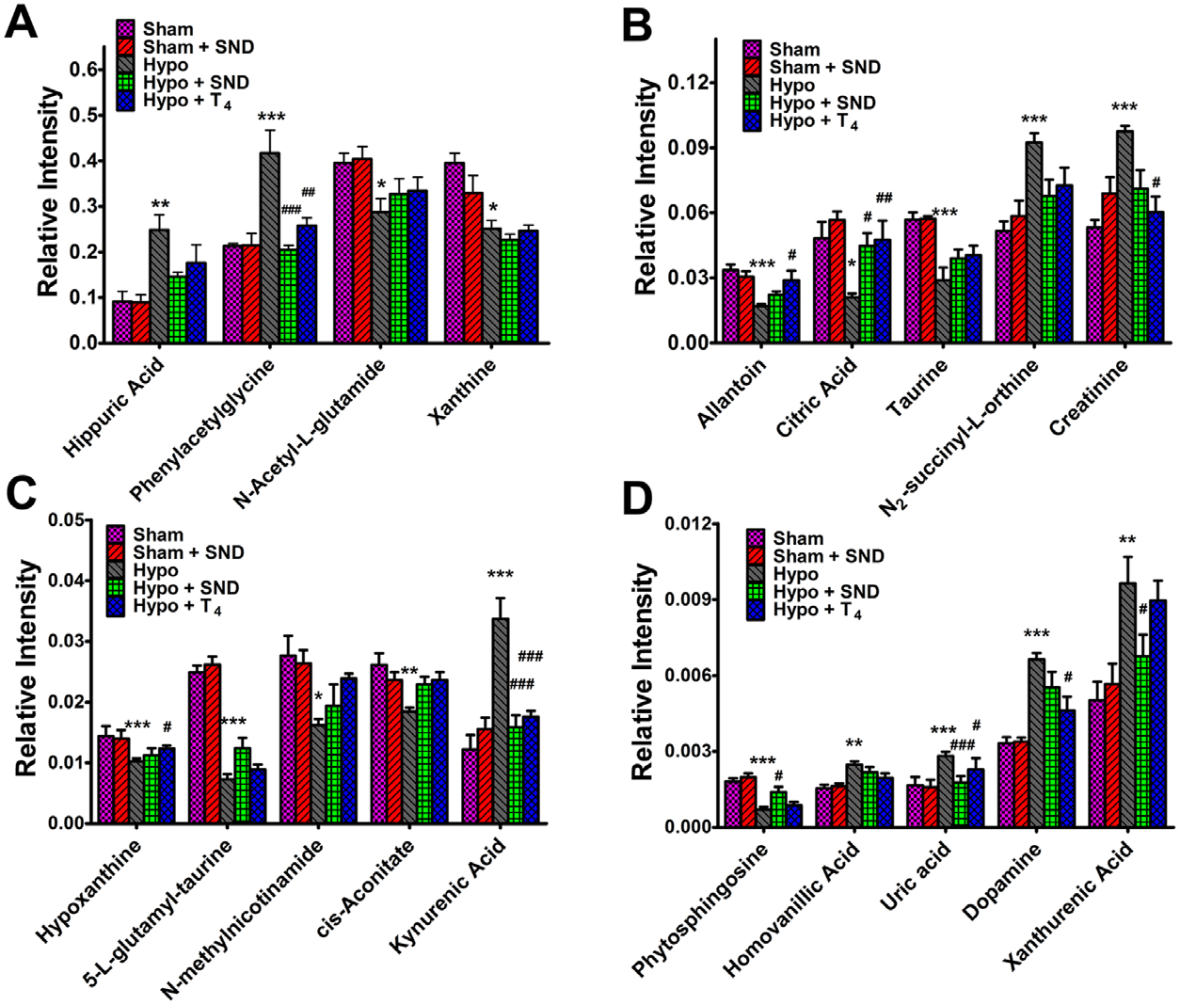
Comparison of different ions in urine of Sham group, Sham + *SND* group, Hypo group, Hypo + *SND* group and Hypo + T_4_ group after 4-week treatment. * *p*-Value<0.05 compared to Sham group, ** *p*-Value< 0.01 compared to Sham group, *** *p*-Value<0.001 compared to Sham group; ^#^ *p*-Value<0.05 compared to Hypo group, ^##^ *p*-Value<0.01 compared to Hypo group, ^###^ *p*-Value<0.001 compared to Hypo group.

To further compare the recovery condition of the 19 potential biomarkers by *SND* and T_4_, the relative intensity of the 19 biomarkers (the relative peak areas of the 19 biomarkers to their respective total integrated area of the spectra) between the five surgical groups were tested by ANOVA and the Tukey post hoc test for comparisons of multiple groups. The detailed data of relative intensity of 19 biomarkers in five surgical groups are given in **Table S3**. Compared to Hypo group, 6 of the 19 biomarkers including phenylacetylglycine, citric acid, kynurenic acid, phytosphingosine, uric acid and xanthurenic acid were significantly reversed by *SND*; while 8 of 19 biomarkers including phenylacetylglycine, allantoin, citric acid, creatinine, hypoxanthine, kynurenic acid, uric acid and dopamine were markedly restored by positive drug T_4_ (**Figure 6**). The other biomarkers were also regulated back to the relative normal status in varying degrees, except for xanthine. The results demonstrated that *SND* and T_4_ may play their therapeutical effects on hypothyroidism though various pathways.

## Discussion

Hypothyroidism is a kind of complex disease and the symptoms present in more than one issue and even spread to the whole body. The dominant treatment for hypothyroidism is thyroid hormone replacement therapy at present because of its certain efficiency. However, it may largely focus on thyroid per se, but overlook the complexity of disease, integrity of organism, and the potential side effects to some extent. While TCM, a complex medical science, places the human body into a large system for observation and adjusts the human body to remain in a healthy status [38]. Therefore, key insights from this point of integrating western medicine and the core thinking of Chinese medicine may shed a new light on the treatment of hypothyroidism. In the concept of TCM, hypothyroidism belongs to the scope of “consumptive disease” or “asthenic disease”, and the essential pathogenesis of hypothyroidism is “Kidney-*Yang* Deficiency Syndrome”. Here, kidney is more than the urinary organ, but represents the foundation of every life activity in human body.

Modern medicine research indicates that the functional disorder of hypothalamic-pituitary-target gland (adrenal, thyroid and gonad) has a tight relationship with “Kidney-*Yang* Deficiency Syndrome”. The Chinese medicine *SND*, one typical example of multicomponent medicine, is commonly applied for “Kidney-*Yang* Deficiency Syndrome” [20]. Especially, it is suitable to treat hypothyroidism, which is one of the most representive diseases caused by “Kidney-*Yang* Deficiency Syndrome”. Nevertheless, its holistic mechanistic understanding on hypothyroidism is still insufficient.

**Figure 7:**
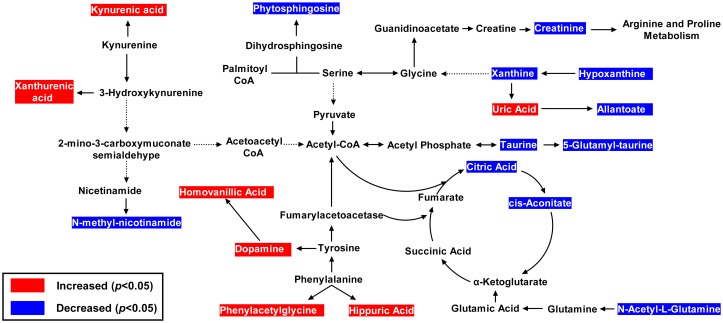
The integrative plot of the metabolites and the relevant pathways changing for thyroidectomy-induced hypothyroidism in circulation system. Metabolites with red dashed area present significant increase in Hypo group compared to Sham group. Metabolites with blue dashed area present significant decrease in Hypo group compared to Sham group.

Three typical hypothyroidism models (MMI-, PTU- and thyroidectomy-induced hypothyroidism) were applied to characterize the metabolic changes of hypothyroidism comprehensively. Among the selected biomarkers related with the three models, nine of them shared in all the three models, including N_2_-succinyl-L-ornithine, kynurenic acid, xanthurenic acid, phytosphingosine, phenylacetylglycine, citric acid, taurine, hypoxanthine and hippuric acid. The three models are all involved in energy metabolism, amino acid metabolism and sphingolipid metabolism. It is found that more biomarkers were selected related to phenylalanine metabolism in MMI-induced hypothyroidism model and purine metabolism played a specific role in thyroidectomy-induced hypothyroidism model. The detailed comparison of biomarkers and pathways for MMI- and PTU-induced hypothyroidism models is showed in **Figure S1**. **Figure 7** depicts the integrative plot of the metabolites and the relevant pathways changing for thyroidectomy-induced hypothyroidism.

In this study, a global urine metabolomic analysis based on UHPLC-TOF/MS was applied to provide comprehensive and complementary insights into hypothyroidism at the metabolic level. Based on the 19 selected biomarkers related to thyroidectomy-induced hypothyroidism model, the therapeutical effects of *SND* was assessed and compared with positive drug T_4_. The results demonstrated that both *SND* and T_4_ can improve the thyroid dysfunction with different degrees, by partially regulating energy metabolism, sphingolipid metabolism, amino acid metabolism, and purine metabolism.

### Biochemical interpretation

It has been well documented that *cis*-aconitate, succinic acid, citric acid and α-ketoglutaric acid are the vital substances of tricarboxylic acids cycle (TCA), which is the main pathway of glucose degradation and the primary energy supplier for universal organisms. In this study, these four pivotal intermediates of TCA cycle were significantly decreased in the hypothyroid group compared to the control group, suggesting that hypothyroidism may inhibit the TCA cycle markedly and ultimately reduce energy metabolism. It has been reported that the thyroid hormones influence the activity of TCA cycle and thyroid hormone-induced increase of mitochondrial Ca^2+^ and substrate supply can stimulate the overall TCA cycle activity [39]. A wealth of evidence further supports that hypothyroidism remarkably reduces the biogenesis and respiratory capacity in free mitochondria and neuronal oxygen consumption in the cerebral cortex in developing rats [40]. The decreased level of TCA cycle in our study indicated that hypothyroidism was related with energy metabolism dysfunction, which quite agreed with the results of previous studies about thyroid disorder [18], [33], [41].

Dihydrosphingosine and phytosphingosine, two ceramide-related metabolites detected in this study, were dramatically decreased, indicating that sphingolipid metabolism turned to be abnormal in response to hypothyroidism. These two metabolites play important roles in sphingolipids biosynthesis and metabolism, and convert into ceramide [42]. In our study, the reduced pool size of dihydrosphingosine and phytosphingosine consequently resulted in a decrease in ceramide. Ceramide is the vital second messenger in the sphingomyelin signaling pathway and its content is regulated by the thyroid hormone [43]. Gorska.J *et al* found that hypothyreosis reduced the total content of ceramide in rat tissues [44]. Precious studies showed that PTU-induced hypothyroidism would affect the synthesis of sphingomyelin in chick liver microsomes [45], which is consistent with our observation that sphingolipid metabolism was also involved in hypothyroidism.

In our study, the abnormal variations of phenylacetylglycine, dopamine, phenylethylamine, hippuric acid and homovanillic acid suggested that phenylalanine metabolism was greatly disturbed by hypothyroidism. These potential biomarkers are the metabolites of phenylalanine and tyrosine. It is well known that phenylalanine can be converted firstly to tyrosine by phenylalanine hydroxylase, and then to catecholamines (e.g. dopamine, norepinephrine and epinephrine) [46]. When the hypothyroidism models were made in the study, the pathway of phenylalanine may be partly blocked, in which metabolites of phenylalanine and tyrosine were excreted to urine increasingly. It has been reported that CSF levels of phenylalanine and tyrosine are closely related to thyroid function [47] and neonatal hypothyroidism induces striatal dopaminergic dysfunction [48]. From these findings, we can infer that hypothyroidism may be involved in phenylalanine metabolism.

Tryptophan is an essential amino acid and serves a variety of roles in metabolism. Hypothyroidism patients commonly have symptoms of anorexia and poor appetite, it is reported that a dietary tryptophan deficiency can alter thyroid hormone levels [49]. In addition, it was observed in our study that kynurenic acid, xanthurenic acid, indole and indoxyl, the metabolic products of tryptophan, ascended considerably in the hypothyroid group compared with those in the control group. The unusual increased catabolism of tryptophan may aggravate the shortage of tryptophan *in vivo*. Some studies have shown that the deficit of tryptophan can lead to thyroid dysfunction [50], [51]. Therefore, the altered contents of kynurenic acid, xanthurenic acid, indole and indoxyl at present may predict the abnormality of tryptophan metabolism in hypothyroid rats.

Taurine is considered to be the second most abundant in the body's muscle after glutamine. Taurine supplementation can play a protective role against the increased oxidative stress resulting from hypothyroidism by raising serum paraoxonase and arylesterase activities [52], [53]. The decreased level of taurine and its metabolic product 5-L-glutamyl-taurine, observed in urine metabolite profiles of hypothyroid group compared with the controls, may be a cue of deregulation of taurine and hypotaurine metabolism in hypothyroidism.

In addition, it is of note that the specific relationship between other disturbed pathways here (e.g. arginine and proline metabolism, purine metabolism etc) and the occurrence of hypothyroidism remains obscure and awaits further investigation by combining with other data (proteomics, transcriptomics and genomics) in the future.

### Mechanism supposition of *SND*

The primary function of thyroid hormone is to run the body's metabolism, so it is understandable that patients with hypothyroidism will have symptoms associated with a slow metabolism, which mainly result from insufficiency of oxygen utilization. It has a close relationship with mitochondria, because the most prominent role of mitochondria is to produce ATP through respiration by utilizing oxygen and to regulate cellular metabolism. It was reported that *SND*, a “*Yang*-invigorating” Chinese tonifying herb, might speed up ATP generation by protecting mitochondria from oxidation injury [54], [55], which often occurs in the hypothyroid condition. It was found in our study that four intermediates of TCA were up-regulated in the Hypo + *SND* group compared to the Hypo group, indicating that *SND* could enhance mitochondrial function and body metabolism.

According to TCM theory, the corn pathogenesis of hypothyroidism is “Kidney-*Yang* Deficiency Syndrome”. The Chinese medicine *SND*, which can recuperate the depleted *Yang* and rescue patients from cases of exhaustion of *Yang*-energy [56], is quite fit for the treatment of hypothyroidism. *SND* is composed of three crude drugs: Acontium carmichaeli, Zingiber officinale and Glycyrrhiza uralensis. Acontium carmichaeli was historically recorded as one of “The Four Pillars” of Chinese herbs, as the principal drug in the famous formula *SND*, invigorating *Yang* of kidney greatly and treating weak diseases in human [57]. It has been documented in the class *Materia medica* that Acontium carmichaeli can play an essential role in regulating the endocrine system of the human body [58]. Zingiber officinale serves as the ministerial drug in *SND*, and its major pharmacological acting appears to be antioxidative, anti-inflammatory and digestive aid [59]. It was reported that the content of aconitum alkaloids in Acontium carmichaeli increased 36.40% when Acontium carmichaeli was used with Zingiber officinale [60], which may provide evidence to the speculation that Zingiber officinale plays an assistant role in strengthening the efficacy of Acontium carmichaeli. Glycyrrhiza uralensis, as a tonifying herbal medicine, serves as the adjunctive and messenger drug in *SND*. It is found that when Acontium carmichaeli and Glycyrrhiza uralensis are mixed in the form of a decoction, Glycyrrhiza uralensis can decrease the toxicity of Acontium carmichaeli and make the effect of invigoration last for an even longer time [57].

The combination of different types of medical herbs above in *SND* can benefit from each other with different roles in the formula, and ultimately gain the goal of enhancing efficacy and reducing toxicity, which quite caters to the core thinking of TCM theory. Our study demonstrated that *SND* had a time-related intervention effect on hypothyroidism, which may provide complementary insights into the conventional thyroid hormone replacement therapy on hypothyroidism.

In summary, a metabonomic method based on UHPLC-Q-TOFMS was developed to study the metabolic changes in hypothyroid rats and the intervention effect of TCM *SND*. Three typical rat models of hypothyroidism were applied to characterize the metabolic changes of hypothyroidism comprehensively. 17, 21 and 19 potential biomarkers were identified related to MMI-, PTU- and thyroidectomy-induced hypothyroidism, respectively, primarily involved in energy metabolism, sphingolipid metabolism, amino acid metabolism, purine metabolism and so on. Using the potential biomarkers found in this study as the criteria, we found that both *SND* and positive drug T_4_ had obvious recovery effects on hypothyroidism through reversing partial disturbed metabolic pathways. However, due to the small number of rats in this study, this pilot study is more like to demonstrate the methodology rather than to provide definitive conclusions about the disease and drug. Application of these traces in other species including humans and combination with other “omics” strategies to validate hypothyroidism-related metabolite changes and value the holistic efficacy of *SND* await further study. Our results show that the metabonomic platform gains the potential to shed new light on the hypothyroidism-related biomarkers and pathophysiology, and provides a complementally effective treatment on hypothyroidism with TCM *SND*.

## Materials and Methods

### Ethics Statement

All animal experiments were approved by the Administrative Committee of Experimental Animal Care and Use of Second Military Medical University (SMMU, Licence No. 2011023), and conformed to the National Institute of Health guidelines on the ethical use of animals.

### Reagents and Materials

Triiodothyronine radioimmunoassay kit and thyroxin radioimmunoassay kit were purchased from Beijing North Institute of Biological Technology (Beijing, China). HPLC-grade methanol and acetonitril (ACN) were purchased from Merck (Darmstadt, Germany). Formic acid was obtained from Fluka (Buchs, Switzerland). Ammonium formate, PTU, MMI, ketamine hydrochloride, xylazine, acepromazine, ketorolac, gentamicin and standard kynurenic acid, homovanillic acid were purchased from Sigma-Aldrich (St Louis, MO, USA). Dihydrosphingosine and phytosphingosine were purchased from Acros Organics (NewJersey, USA). Spermidine, creatine, creatinine, xanthurenic acid, citric acid, succinic acid, α-ketoglutaric acid, taurine, allantoin, uric acid, and hippuric acid were obtained from Shanghai Jingchun Reagent Co. Ultrapure water was prepared with a Milli-Q water purification system (Millipore, Bedford, MA, USA).

*Acontium carmichaeli* (from Sichuan, China), *Glycyrrhiza uralensis* (from Xinjiang, China) and *Zingiber officinale* (from Guizhou, China) were purchased from Lei Yunshang Medicine Corp. (Shanghai, China). They were authenticated by Lianna Sun (Department of Pharmacognosy, School of Pharmacy, Second Military Medical University, Shanghai, China) and met the standards recorded in Chinese Pharmacopoeia (2010 Edition).

### Preparation of *SND* and phytochemical investigation

According to the original composition of *SND* recorded in Chinese Pharmacopoeia 2010 edition, *SND* was prepared using the following procedure. The crude drugs of *A. carmichaeli* 90 g, *Z.officinale* 60 g and *G. uralensis* 90 g were immersed in 2.4 liter water for 1 h and then decocted to boil for 2 h. The decoction was filtered through four layers of gauze. Next, the dregs were boiled once again for 1 h with 1.9 liters of water and the decoction was filtrated out with the above method. Afterward, the successive decoctions were merged and condensed under decompression. Finally, the extraction solution was made to a concentration of 1.0 g crude drugs/mL. According to our previous published paper [61], 53 components of *SND* were identified. Parallel *SND* extraction experiment showed that RSD of typical components' peak area was less than 4%, indicating the reproducibility of *SND* extracts was good.

### Hypothyroidism models and Treatment

The study was approved by the national legislation of China and local guidelines and performed at the Center of Laboratory Animals of the Second Military Medical University (Shanghai, China). 64 male Wistar rats (170±15 g) commercially obtained from the Slac Laboratory Animal Co., LTD (Shanghai, China) were maintained under standard laboratory conditions (temperature of 21–23°C, relative humidity 45–65%, and 12 h/12 h light/dark cycle) with aseptic food and tap water *ad libitum*. After one-week habituation, all animals were housed individually in metabolism cages and allowed to acclimatize for additional 24 h.

The rats were randomly divided into eight groups (8 rats/group). A. antithyroid drug-induced hypothyroid groups consisted of three groups: (1) the control group, which were treated with normal tap water for 4 weeks, (2) MMI-induced hypothyroid group (MMI group), which received 0.04% MMI (wt/vol) in the drinking water for 4 weeks as previous study described [62], (3) PTU-induced hypothyroid group (PTU group), which received 0.05% PTU (wt/vol) in the drinking water for 4 weeks as described by Srikanta Jena et al [63]. The dose of antithyroid drugs (MMI and PTU) was adjusted according to water intake and body weight every 3 days throughout the experiment, as described before [64]. B: surgery-induced hypothyroid groups consisted of five groups: (4) Sham group and (5) Sham + *SND* group, which received false thyroidectomy and postoperative treatment; (6) thyroidectomy-induced hypothyroid group (Hypo group), (7) *SND*-treated thyroidectomized group (Hypo + *SND* group) and (8) T_4_-treated thyroidectomized group (Hypo + T_4_ group), which had the thyroid gland removed and postoperative treatment.

The thyroidectomy was done in rats anesthetized with ketamine (10 mg/kg, intramuscular; i.m.) and xylazine (5 mg/kg, i.m.), as previously described [65]. Briefly, a midline skin incision was made along the length of the neck. The underlying tissues were cleared and the salivary glands were retracted laterally. The two halves of the sternohyoid muscle were separated and retracted laterally. Thyroid muscle was separated from the thyroid gland lobes and retracted along with the sternohyoid muscle. A midline cut was made in the isthmus and the thyroid glands were excised bilaterally. Extreme care was taken so as not to damage the laryngeal nerve. Sham rats underwent a surgical procedure in which the rats were anesthetized, the trachea was exposed and the incision was closed, simulating thyroidectomy. After surgery, ketorolac (50 mg/kg, i.m.) and gentamicin (10 mg/kg) were administered over 5 days to alleviate pain and prevent infection. Rats were monitored closely for at least 1 week after the surgery for complications and animals were used for treatment 4 weeks later.

During the phase of treatment, animals in the Sham + *SND* group and Hypo + *SND* group received *SND* by oral gavage at a daily dose of 10 g/kg body weight (equal to 10 mL/kg body weight) for the following 4 weeks. Animals in Hypo + T_4_ group were administered levothyroxin 20 ug/kg/day i.p. for 4 weeks according to previous study [65]. And animals in the Sham and Hypo groups were administered with saline for 4 consecutive weeks.

### Sample collection and preparation

Throughout the animal study, all 64 rats were weighted twice a week to monitor the progress of hypothyroidism and evaluate the effectiveness of *SND* on hypothyroidism. The rectal temperature, food intake and water intake were recorded before and after hypothyroidism model established in control, MMI and PTU groups, and these three physical variables were measured in surgical groups before surgery, 4 weeks after surgery and 4 weeks after treatment. The blood was collected from the suborbital vein before the hypothyroidism model established, at the end of hypothyroidism model established and at the end of treatment. Serum biochemical parameters including total triiodothyronine (T_3_) and total thyroxin (T_4_) were determined by radioimmunoassay. Samples of 24-h urine were collected before the hypothyroidism model established, at the end of hypothyroidism model established in all animals and at the designed time intervals during treatment: at week 2, 3 and 4 after treatment in the surgical groups. Sodium azide was added to the collection vessels as an antibacterial agent. After centrifugation at 4000 rpm for 15 min at 4°C to remove particle contaminants, the supernatants were immediately stored in aliquots at −80°C before UHPLC-Q-TOFMS analysis.

The urine samples were thawed at room temperature prior to analysis. To reduce the effect of the solvent and get a good peak shape, 100 uL urine sample was diluted with 400 µL acetonitrile and centrifuged at 15,000 g for 10 min at 4°C [35]. The clear supernatant was transferred to an autosampler vial and kept at 4°C. A 4 uL aliquot was made onto the column in each run. An in-house quality control (QC) was prepared by pooling and mixing the same volume of each sample. The QC sample was run prior to the start of the analytical run for six times to “condition” the system and analyzed after every 8 samples to check for system stability [34]. Seven parallel samples obtained from a random urine sample were extracted by the same extraction method and injected continuously to evaluate repeatability of method.

### UHPLC-MS analysis

UHPLC-MS analysis was performed on Agilent 1290 Infinity LC system coupled to Agilent 6530 Accurate-Mass Quadrupole Time-of-Flight (Q-TOF) mass spectrometer (Agilent, USA). Chromatographic separations were performed on an ACQUITY UPLC HSS T3 column (2.1 mm × 100 mm, 1.8 µm, Waters, Milford, Ireland) maintained at 45°C. The mobile phase consisted of 0.1% formic acid (A) and ACN modified with 0.1% formic acid (B). The following gradient program was used: 0%B at 0–2 min, 0%–15% B at 2–10 min, 15%–30% B at 10–14 min, 30%–95% B at 14–17 min, 95% B at 17–19 min, 95%-0%B at 19–20 min and followed by re-equilibrated step of 4 min. The flow rate was 400 ml/min and the injection volume was 4 µl.

Mass spectrometry was performed on an Agilent 6530 Accurate-Mass Quadrupole Time-of-Flight (Q-TOF) mass spectrometer (Agilent, USA), operating in both positive and negative ion modes. The capillary voltage was 3.5 kV, drying gas flow was 11 L/min, and the gas temperature was 350°C. The nebulizer pressure was set at 45 psig. The fragmentor voltage was set at 120 V and skimmer voltage was set at 60 V. All analyses were acquired using a mixture of 10 mM purine (*m/z* 121.0508) and 2 mM hexakis phosphazine (*m/z* 922.0097) as internal standards to ensure mass accuracy and reproducibility. Data were collected in a centroid mode and the mass range was set at *m/z* 50–1000 using extended dynamic range. MS/MS analysis was carried out to study the structure of potential biomarkers. MS spectra were collected at 2 spectra/s, and MS/MS spectra were collected at 0.5 spectra/s, with a medium isolation window (∼4 *m/z*) and the collision energy was 18 V.

### Data analysis

First, raw data in the instrument specific format (.d) was converted to common data format (.mzdata) files by a conversion software program, in which the isotope interferences were excluded. The program XCMS (http://metlin.scripps.edu/download/) was applied for peak alignment of the data in the time domain and automatic integration and extraction of the peak intensities [66]. XCMS parameters were default settings except that the full width at half-maximum (FWHM) was set to 10 and the retention time window (BW) was set to 9. Metabolites that did not exist in 80% of the samples in either group were filtered [67]. The retention time and *m/z* data pair were exported to an Excel table (Microsoft). Before the multivariate data analysis, for each chromatogram, the intensity of each ion was normalized to the total ion intensity, in order to partially compensate for the concentration bias of metabolites between samples and to obtain the relative intensity of metabolites.

The resulting normalized data were exported to SIMCA-P V12.0.0 Demo (Umetrics, Sweden) for partial least-squares-discriminate analysis (PLS-DA) after mean-centering and pareto scaling. The quality of the model was evaluated by the cross-validation parameter Q^2^, R^2^Y. One-way ANOVA was performed to reveal the statistical differences in the significance of variation among Sham, Sham + *SND*, Hypo, Hypo + *SND* and Hypo + T_4_ groups. And the Tukey post hoc test was applied for comparisons of multiple groups. The differences were considered significant when *p*<0.05. To account for multiple hypothesis testing, the false discovery rate was estimated as the maximum q value in the set of significant differences for the metabonomic data set. False discovery rates were computed using the R package q value (http://www.r-project.org/). The significance of differences between the groups was evaluated by the *p* value for the fixed-effect parameter estimate of group differences.

## Supporting Information

Figure S1 **Comparison of biomarkers and related pathways between MMI- and PTU-induced hypothyroidism models.**
(TIF)

Table S1 **Sample determination of total triiodothyronine (T_3_) and total thyroxine (T_4_) in rat serum for the antithyroid drug-induced hypothyroid groups (mean ± S.D.) (n = 8).**
(DOC)

Table S2 **The physical variance for the antithyroid drug-induced hypothyroid groups (mean ± S.D.) (n = 8).**
(DOC)

Table S3 **Relative intensity of potential biomarkers in thyroidectomy-induced hypothyroid rat urine.**
(DOC)
