# Supplementary figures and images for: Metabolic Profiling Provides a System Understanding of Hypothyroidism in Rats and Its Application
Source: PLoS One. 2013 Feb 7;8(2):e55599. doi: 10.1371/journal.pone.0055599 (PMC3567130; doi:10.1371/journal.pone.0055599)

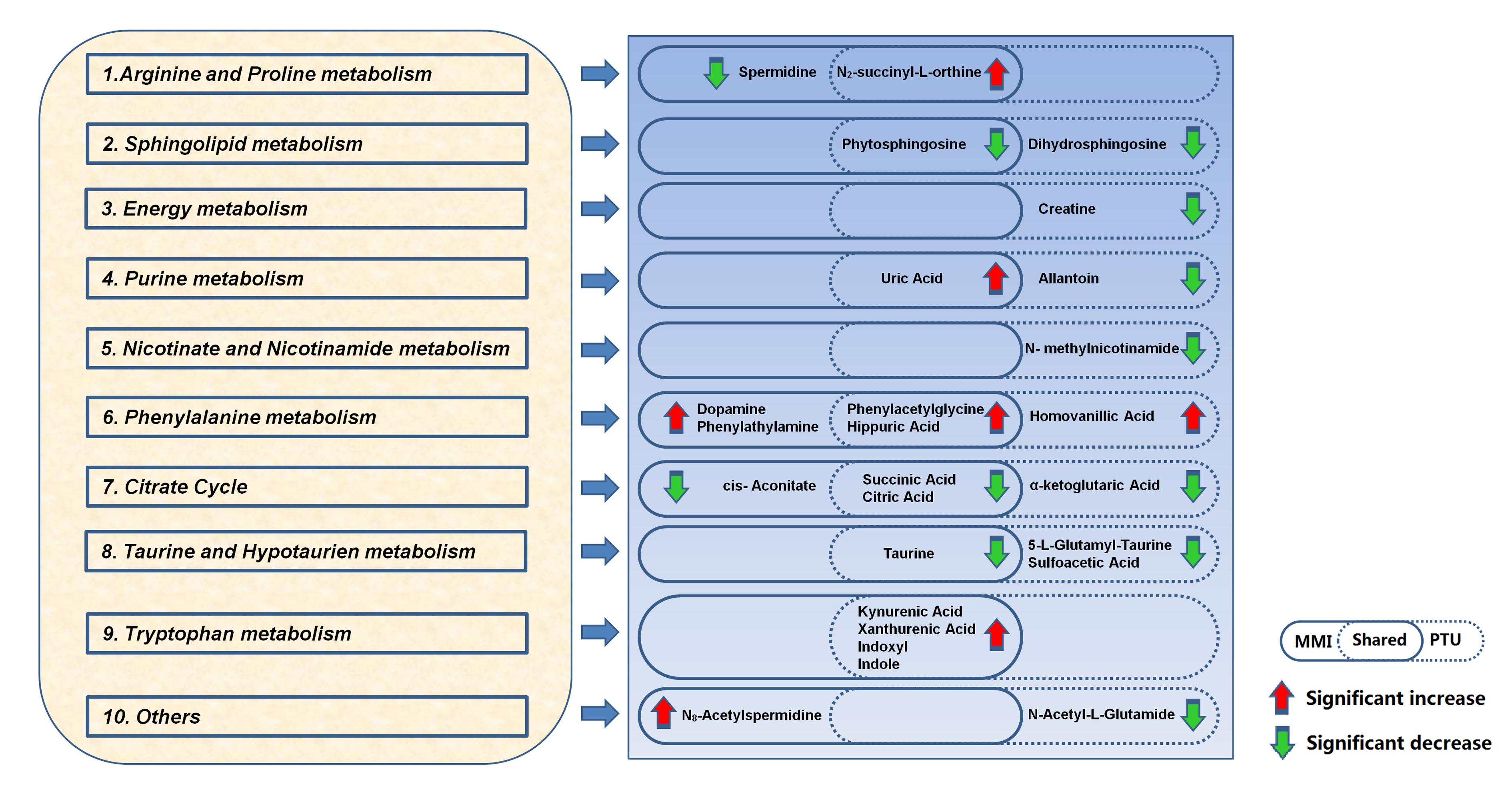

Supplement: Figure S1 — Comparison of biomarkers and related pathways between MMI- and PTU-induced hypothyroidism models. (TIF) [file pone.0055599.s001.tif]
