# Supplementary material for: Metabolic Profiling Provides a System Understanding of Hypothyroidism in Rats and Its Application
Source: PLoS One. 2013 Feb 7;8(2):e55599. doi: 10.1371/journal.pone.0055599 (PMC3567130; doi:10.1371/journal.pone.0055599)
Supplement: Table S1 — Sample determination of total triiodothyronine (T3) and total thyroxine (T4) in rat serum for the antithyroid drug-induced hypothyroid groups (mean ± S.D.) (n = 8). (DOC) [file pone.0055599.s002.doc]

| **Table S-1. Sample determination of total triiodothyronine (T_3_) and total thyroxine (T_4_) in rat serum for the antithyroid drug-induced hypothyroid groups (mean±S.D.)*^a^* (n=8)** | | | | | |
| --- | --- | --- | --- | --- | --- |
| Group | T_3_ | |  | T_4_ | |
|  | Before model established | After model established |  | Before model established | After model established |
| Control group | 0.47±0.07 | 0.48±0.08 |  | 48.86±4.32 | 47.45±4.13 |
| MMI group | 0.48±0.06 | 0.18±0.03* |  | 47.97±4.12 | 9.59±1.37* |
| PTU group | 0.47±0.06 | 0.21±0.03* |  | 49.13±3.96 | 11.82±1.85* |
| *^a^* The unit of T_3_ and T_4_ is ng/mL | | | | |  |
| * *p*<0.001 (compared to Control group) | | | | | |
