# Supplementary material for: Metabolic Profiling Provides a System Understanding of Hypothyroidism in Rats and Its Application
Source: PLoS One. 2013 Feb 7;8(2):e55599. doi: 10.1371/journal.pone.0055599 (PMC3567130; doi:10.1371/journal.pone.0055599)
Supplement: Table S2 — The physical variance for the antithyroid drug-induced hypothyroid groups (mean ± S.D.) (n = 8). (DOC) [file pone.0055599.s003.doc]

| **Table S-2 The physical variance for the antithyroid drug-induced hypothyroid groups (mean±S.D.) (n=8)** | | | | | | | | |
| --- | --- | --- | --- | --- | --- | --- | --- | --- |
| Group | Rectal Temperature ( ℃ ) | |  | Food Intake (g/day) | |  | Water Intake (g/day) | |
|  | Before model established | After model established |  | Before model established | After model established |  | Before model established | After model established |
| Control group | 37.7±0.2 | 37.8±0.3 |  | 24.3±1.8 | 24.8±1.8 |  | 25.2±2.2 | 25.8±2.3 |
| MMI group | 37.8±0.3 | 37.1±0.1** |  | 24.4±1.9 | 18.7±1.7*** |  | 25.6±2.4 | 22.7±2.1** |
| PTU group | 37.6±0.2 | 37.4±0.2* |  | 24.6±2.0 | 19.8±1.9*** |  | 25.7±2.1 | 23.1±2.2* |
| * *p*<0.05, ** *p*<0.01, *** *p*<0.001 compared to Control group | | | | | | | | |
