# Supplementary material for: Metabolic Profiling Provides a System Understanding of Hypothyroidism in Rats and Its Application
Source: PLoS One. 2013 Feb 7;8(2):e55599. doi: 10.1371/journal.pone.0055599 (PMC3567130; doi:10.1371/journal.pone.0055599)
Supplement: Table S3 — Relative intensity of potential biomarkers in thyroidectomy-induced hypothyroid rat urine. (DOC) [file pone.0055599.s004.doc]

**Table S-3 Relative intensity of potential biomarkers in thyroidectomy-induced hypothyroid rat urine**

| NO | Identification | Sham group (Mean ± S.D.) | Sham + *SND* group (Mean ± S.D.) | Hypo group (Mean ± S.D.) | Hypo + *SND* group (Mean ± S.D.) | Hypo + T_4_ group (Mean ± S.D.) |
| --- | --- | --- | --- | --- | --- | --- |
| 1 | N_2_-succinyl-L-ornithine | 0.0516±0.0044 | 0.0583±0.0072 | 0.0924±0.0042 | 0.0676±0.0076 | 0.0726±0.0083 |
| 2 | Kynurenic Acid | 0.0122±0.0023 | 0.0155±0.0018 | 0.0337±0.0033 | 0.0158±0.0020 | 0.0175±0.0009 |
| 3 | Xanthurenic Acid | 0.0050±0.0007 | 0.0056±0.0008 | 0.0096±0.0010 | 0.0056±0.0003 | 0.0067±0.0008 |
| 4 | Phytosphingosine | 0.0018±0.0001 | 0.0019±0.0001 | 0.0007±0.0001 | 0.0013±0.0002 | 0.0009±0.0001 |
| 5 | N-methylnicotinamide | 0.0276±0.0032 | 0.0264±0.0021 | 0.0162±0.0010 | 0.0193±0.0036 | 0.0238±0.0008 |
| 6 | Creatinine | 0.0532±0.0033 | 0.0688±0.0076 | 0.1037±0.0220 | 0.0712±0.0085 | 0.0603±0.0071 |
| 7 | Phenylacetylglycine | 0.2132±0.0055 | 0.2147±0.0258 | 0.4171±0.0503 | 0.2048±0.0102 | 0.2576±0.0176 |
| 8 | Dopamine | 0.0033±0.0002 | 0.0033±0.0001 | 0.0066±0.0002 | 0.0055±0.0006 | 0.0046±0.0005 |
| 9 | cis-Aconitate | 0.0262±0.0018 | 0.0236±0.0013 | 0.0184±0.0006 | 0.0229±0.0012 | 0.0236±0.0013 |
| 10 | Citric Acid | 0.0483±0.0073 | 0.0566±0.0040 | 0.0208±0.0019 | 0.0446±0.0059 | 0.0476±0.0086 |
| 11 | Taurine | 0.0568±0.0033 | 0.0572±0.0013 | 0.0288±0.0058 | 0.0390±0.0039 | 0.0403±0.0045 |
| 12 | 5-L-Glutamyl-Taurine | 0.0249±0.0011 | 0.0262±0.0012 | 0.0072±0.0008 | 0.0124±0.0016 | 0.0088±0.0008 |
| 13 | Allantoin | 0.0335±0.0025 | 0.0303±0.0025 | 0.0168±0.0010 | 0.0221±0.0016 | 0.0289±0.0043 |
| 14 | Uric Acid | 0.0013±0.0002 | 0.0016±0.0002 | 0.0034±0.0001 | 0.0017±0.0002 | 0.0023±0.0002 |
| 15 | Hypoxanthine | 0.0144±0.0016 | 0.0139±0.0013 | 0.0074±0.0001 | 0.0112±0.0011 | 0.0123±0.0005 |
| 16 | Xanthine | 0.3954±0.0222 | 0.3298±0.0374 | 0.2511±0.0182 | 0.3271±0.0337 | 0.3345±0.0298 |
| 17 | Hippuric Acid | 0.0912±0.0225 | 0.0887±0.0176 | 0.2484±0.0331 | 0.1447±0.0111 | 0.1761±0.0399 |
| 18 | Homovanilic Acid | 0.0015±0.0001 | 0.0016±0.0001 | 0.0024±0.0001 | 0.0021±0.0001 | 0.0019±0.0001 |
| 19 | N-Acetyl-L-Glutamide | 0.3954±0.0222 | 0.4048±0.0265 | 0.2877±0.0290 | 0.3271±0.0337 | 0.3345±0.0298 |
